# Political economies and environmental futures for the sharing economy

**DOI:** 10.1098/rsta.2016.0367

**Published:** 2017-05-01

**Authors:** Koen Frenken

**Affiliations:** Innovation Studies, Copernicus Institute of Sustainable Development, Utrecht University, PO Box 80115, 3508TC Utrecht, The Netherlands

**Keywords:** sharing economy, circular economy, access economy, peer-to-peer markets, sustainable consumption, collaborative consumption

## Abstract

The sudden rise of the sharing economy has sparked an intense public debate about its definition, its effects and its future regulation. Here, I attempt to provide analytical guidance by defining the sharing economy as the practice that consumers grant each other temporary access to their under-utilized physical assets. Using this definition, the rise of the sharing economy can be understood as occurring at the intersection of three salient economic trends: peer-to-peer exchange, access over ownership and circular business models. I shortly discuss some of the environmental impacts of online sharing platforms and then articulate three possible futures of the sharing economy: a capitalist future cumulating in monopolistic super-platforms allowing for seamless services, a state-led future that shifts taxation from labour to capital and redistributes the gains of sharing from winners to losers, and a citizen-led future based on cooperatively owned platforms under democratic control. The nature and size of the social and environmental impacts are expected to differ greatly in each of the three scenarios.

This article is part of the themed issue ‘Material demand reduction’.

## 1. Introduction

Among the most significant economic developments over the past 10 years has been the rise of the sharing economy. With the advent of online platforms, many consumers have started to borrow or rent goods from other consumers. At the same time, institutions are being challenged as consumers on such platforms do not adhere to the market regulations and tax obligations that apply to regular rental businesses. This has led to widespread controversy about the desirability of sharing platforms and to what extent current regulations should be adapted [1].

From an analytical perspective, the sharing economy can be defined in a materialistic sense in that consumers share a physical artefact in its *usage*. The meaning of sharing as ‘sharing is use’ is helpful if one wants to focus attention to the environmental benefits. In many cases, consumers gain access to *under-utilized* goods when they rent one another's goods. If sharing practices were to scale up, the total number of consumer goods could go down considerably without loss of consumer welfare. A materialistic definition of sharing thus leaves open whether sharing takes place online or offline and whether sharing is done for free or for a fee [2].

Using this definition, I will reflect on the possible environmental gains that can be expected from the sharing economy. The key argument that is developed in this essay holds that the environmental impacts of the sharing economy will depend critically on the institutions that will regulate and further shape the socio-technical infrastructure underlying the sharing economy. The way online sharing platforms will be governed, how sharing practices will be regulated, and what kind of tax regime will emerge are fundamentally open questions for the future jointly shaped by businesses, citizens, and governments at local, national and transnational scales [1,3,4].

Below, I will highlight institutional issues surrounding data privacy, tax, consumer protection, employee status and platform ownership. Choices for particular institutions for each of these issues are likely to be intertwined and, in particular configurations, to be complementary. This leads me to conceptualize three future political economies of sharing each based on a different institutional order: (i) a platform-capitalistic future of seamless consumption cumulating in monopolistic super-platforms, (ii) a platform-redistributive future where governments shift taxes from platform labour and usage of goods to platform capital and ownership of goods, and (iii) a platform-cooperativist future led by citizens owning and governing online sharing platforms. All three futures will not just differ in the environmental impacts that can be expected to occur, but also in the distribution of economic gains and political power in a future economy organized principally by online platforms.

## 2. Defining the sharing economy

Following a review of the term sharing [5], the most common understanding of sharing is as ‘dividing’ and ‘breaking up in parts’. In this ordinary sense, sharing is about distribution, for example, dividing food among people. Sharing in a distributional sense is a zero-sum game [5]. If one is given more, others will be given less. Sharing economy platforms, by contrast, escape this zero-sum logic. On such platforms, people offer *under*-utilized consumer goods such as cars, houses, parking space, rill, clothing, computer memory, etc. Such ‘shareable goods’ [6] are goods that consumers do not consume all the time and thus provide them with excess capacity. Given this excess capacity, consumers have the opportunity to lend out or rent out their goods at times they do not consume them, without any additional investment. Hence, by lending out or renting out such goods when under-utilized, consumers enter a positive-sum game.

A definition that captures the meaning of sharing in terms of access to someone's consumer good, is the sharing economy as ‘consumers granting each other temporary access to their under-used physical assets (‘idle capacity’), possibly for money’ [2,7]. Note that the meaning of sharing as ‘dividing’ is still preserved. However, it is a good's capacity for usage that is divided among the owner and fellow consumers, once the owner of a good grants ‘peers’ access to a good. Sharing generally involves granting access to an entire good for a particular period of time (e.g. car sharing or home sharing allowing different consumer to use an entire car or house at different times), but sharing can also entail access to parts of a good (e.g. carpooling where a peer is granted one seat in a car) [6].

Sharing here is defined in a ‘materialistic’ sense: sharing takes place once an owner grants other consumer access to a *physical* asset.^1^ That is why sharing in this sense does not pre-specify the kind of social relation that is involved in this practice. Whether or not this practice is governed by monetary transactions is irrelevant for the material side of matters: in both cases sharing leads to a better utilization of an under-utilized physical asset.^2^ Here, I differ from Belk's [9,10] notion of ‘true’ sharing as only those forms of exchange where money does not change hands, which excludes consumers renting out their goods from the sharing economy.

A materialistic definition also implies that a distinction has to be made between consumers granting each other access to their own goods versus consumers providing each other with personal services. Though it has been common to include peer-to-peer service platforms such as taxi, education and cooking as part of the sharing economy [1,11], the key resource that providers offer in such exchanges is immaterial (time and skills). Hence, it makes sense to treat peer-to-peer service provision as fundamentally different from peer-to-peer goods sharing.

## 3. Sharing in context

The definition of sharing as ‘consumers granting each other temporary access to their under-utilized physical assets (‘idle capacity’), possibly for money’, implies that the sharing economy has existed as long as humanity. Hence, sharing should not be equated with sharing using online platforms [12]. Offline, sharing has always been common among family, friends and neighbours. These are the trusted people due to emotional bonding and past interactions. In such social relations, goods were often lent out for free by social obligation rather than rented out for some price [6,13]. Lending or renting out goods to strangers was uncommon due to a lack of information about the trustworthiness of a stranger. The key change with the advent of Internet platform holds that people start to engage in ‘stranger sharing’ [1]. This has been made possible by online platforms that create trust among sharing strangers, mainly by providing a public review system and by providing micro-insurance. Apart from establishing trust, platforms also provide the matching service. Often, this is done algorithmically based on geographical information and user preferences. Once a match is made, a contract and payment is carried out almost fully automatically.^3^ The reason, then, why the online practice of sharing could rise so fast during the past decade lies exactly in the under-utilization aspect of sharing. People share assets they already owned. As a consequence, sharing platforms can scale very fast. The platform only has to provide the information and communications technology (ICT) infrastructure to facilitate sharing, while no party has to make new investments in the physical goods that are being shared. As facilitator, most platforms charge a fee for every transaction rendering their business model potentially very profitable. In many instances, the profit expectation underlies the generous venture capital that platforms received to kick-start the platform, though some also made use of alternative sources of finance (public subsidies, crowdfunding, membership fees).

The definition of sharing economy given above (consumers who grant each other temporary access to their under-utilized physical assets) can be decomposed in three constituting parts. First, the sharing economy is about peer-to-peer exchange or, more precisely in this context, *consumer-to-consumer* interaction. Second, the sharing economy involves temporary *access* either by borrowing or renting. Third, the sharing economy is about a better use of otherwise under-utilized physical assets. All the three constituting parts on their own are instances of broader salient trends in the economy:

— Consumer-to-consumer (c2c) interaction. Consumers offer others access to their consumption goods, and, as such, act as a small rental agency. Consumers taking the role of producers is part of a trend towards prosumerism [14]. When such c2c rental services are transacted through a market with the provider asking money in return, economists now speak of *peer-to-peer economy* [15]. The platform in such market acts as intermediary matching supply and demand and offering auxiliary services such as a ratings, insurances and automatic payment.
— Access rather than ownership. The sharing economy is an instance of the *access economy*, where consumers increasingly opt for access over ownership. Most prominently, car ownership is declining among younger people as access alternatives have been proliferating [16]. Not only can car drivers make use of sharing economy platforms like Relayrides for c2c carsharing or BlaBlaCar for c2c ridesharing, but also of cheap and convenient car rental services (ZipCar, Car2Go, Sixt) or ride-hailing services^4^ (Uber, Lyft, Didi).
— Better use of under-utilized physical assets. In this sense, the sharing economy is an example of the *circular economy*, here simply understood as business models that make efficient use of resources [18]. As more people make use of a single good, fewer goods may be needed to fulfil the same level of demand.

**Figure 1.**
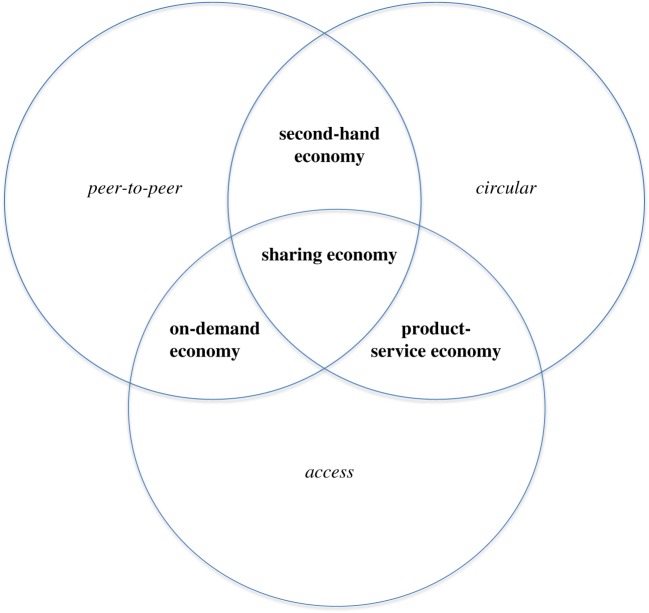
Sharing economy and related types of economy (cf. Frenken *et al*. [2]). (Online version in colour.)

The rapid growth of the sharing economy, then, can be understood in the context of these wider developments unfolding in the economy. The sharing economy occurs at the exact intersection of the aforementioned three trends, with each trend in itself being much more encompassing than just the sharing economy. Once one understands the sharing economy category at the intersection of three more generic types of economic trends, one can also derive the types of economies occurring at the intersection of two of the three trends, as in figure 1 (see also [2]):

— If sharing is based on leasing a good from a company (business-to-consumer, abbreviated b2c) rather than from another consumer, I speak of *product-service economy*. Then, the service consists of the consumer obtaining access to a product, while the company retains ownership. An example is b2c car-rental via Hertz or Zipcar. This type of platform occurs at the intersection of the access economy and circular economy trends.
— Many consumers sell (or give away) their goods to other consumers, which is distinct from sharing goods on a temporary basis, as ownership changes hands. In such cases, I speak of the *second-hand economy*. Second-hand markets are increasingly organized online via platforms such as Ebay or Facebook. This type of platform occurs at the intersection of peer-to-peer exchange and circular economy trends.
— The phenomenon that private individuals deliver services using their time and skills is different from the phenomenon that consumers share their physical assets. A physical asset can be under-utilized by its owner by standing idle, but people cannot [7]. Platforms matching freelancers with consumers are referred to as the *on-demand economy* [19] or gig economy. Examples are platforms for home improvement projects (HomeAdvisor), home cleaning (Helpling) and taxi-rides (‘ride-hailing’) platforms (Uber, Lyft, Didi). Such platforms occur at the intersection of trends towards peer-to-peer exchange and access economy.

Figure 1 is helpful for understanding that some of the platforms cannot be equated one-on-one with the sharing economy, but are better understood as hybrids [2]. For example, on Airbnb one finds people who occasionally rent out their own home when they go away on a trip (sharing economy), but also landlords who own multiple homes and use Airbnb to run an illegal hotel (product-service economy). UberPool is a hybrid in that the driver performs a gig by offering a taxi service (on-demand economy), while the multiple pooling passengers share otherwise under-utilized passenger seats (sharing economy). And through some carsharing platforms one can rent a car from a private individual (sharing economy), but also from the organization itself (product-service economy).

## 4. Sustainable consumption?

The four modes of consumption in figure 1 (sharing, product-service, second-hand, and on-demand) have also been considered a subset of what has become known as ‘collaborative consumption’ [3,11]. The ‘collaborative’ aspect among consumers is always different. In the sharing economy you give someone temporary access to goods; in the second-hand economy this is permanent access to goods; in the on-demand economy, someone delivers a service; and in the b2c product-service economy, a company provides a good to multiple consumers (and consumers indirectly collaborate).

The four instances of collaborative consumption can all be considered examples of sustainable consumption [2]. In each case, it permits consumers to avoid the purchase of a first-hand consumption good, while saving cost at the same time. The total number of goods in the economy can be reduced drastically without giving up consumer welfare. If fewer goods are needed, societies can achieve a reduction in energy use and greenhouse gas (GHG) emissions associated with the production and demolition of goods. Consider the examples of a drill and a car. If a consumer needs a particular physical good, such as a drill, there are four possible platforms: you can buy a drill from an individual (for example on Ebay), you can rent a drill from a company (for example via Home Depot), you can hire someone to drill the hole in your wall (for example on Taskrabbit), or you can ask an individual if you can borrow or rent a drill (for example on Peerby). In all cases, the purchase of a new drill is avoided. For cars, the same logic applies. You can buy a second-hand car (Ebay), you can rent a car via a car-rental company (Avis, Hertz, Car2Go), you can hire an individual to drive you (Lyft, Uber, Didi), or you can rent a car from a private individual (Turo).

Focusing again on the sharing economy, the potential environmental benefit is not just limited to reducing the amount of goods. A second benefit stems from land use efficiency, in particular, fewer parking spaces and empty buildings, which would allow much higher densities of urban living and associated energy efficiencies *per capita* [20]. What is more, sharing provides a resilient infrastructure to serve rare peaks in demand, for example, during mega-events (e.g. Olympic Games), natural disasters (e.g. floods) or humanitarian crises (e.g. refugees). In all these cases, fewer public investments have to be made as to deal effectively with peak demands.

However, to date, there have been few systematic studies on the environmental impact of sharing economy platforms. One reason for this omission is that platforms are reluctant to provide data for independent research given concerns about privacy and trade secrets [4]. Instead, some sharing platforms commissioned their own environmental studies. The world-leading home sharing platform Airbnb [21] had the energy use, water use, GHG emissions and waste production of Airbnb guests compared to hotel guests. The numbers suggest that, compared to hotels, home sharing may generate substantial benefits as Airbnb guests consumed less energy by 63–78%, less water by 12–48% and generated less GHG emissions by 61–89%. Furthermore, waste avoidance was estimated to be 0–32% compared to hotel guests. Ridesharing platform BlaBlaCar (with over 20 million users in Europe) also reported in November 2015 on its environmental impact figures. Its website [22] states that it saved 1 000 000 tons CO_2_ over the past two years roughly equivalent to what 250 000 cars emit every year assuming 4 tons per car per year. The key to this reduction, BlaBlaCar reports, is its higher occupancy rate (2.8 passengers per vehicle) compared to the average occupancy rate (1.7 passengers per car). In both the Airbnb and the BlaBlaCar reports, however, the underlying data nor the methodology have been released, which render the validity of these findings hard to judge.

Different from most sharing economy sectors, quite a number of independent studies exist on the environmental impacts of car sharing. However, these studies have mostly been limited to the b2c variant of car sharing (also known as ‘traditional’ car sharing) where a car sharing organization operates a car fleet that is rented out to local residents (e.g. Zipcar, Car2Go). A recent review [23] suggests that traditional car sharing leads to substantial reductions in car ownership, kilometres driven by car and GHG emissions. However, concerning the latter effect, the exact magnitude is still unclear given the large variance in the estimated reductions in GHG emission ranging from 11% to 51%.

Regarding the more recent trend towards peer-to-peer car sharing where people rent out their own car—fitting our definition of the sharing economy—the environmental impacts remain unclear. As explained before, c2c sharing is a very different business model from the b2c product-service business model. The latter is only profitable in dense urban markets as a business needs sufficient demand for a car to be rented out profitably. In the c2c car sharing model, by contrast, car owners offer their car at zero marginal cost and, hence, such offerings occur both in urban, suburban and rural areas [24]. I know of only one recent study [25] looking at the environmental impacts of both c2c and b2c car sharing. This study found that if one looks at b2c and c2c taken together, 8–13% GHG emission reduction is achieved. The 8–13% is a low number compared to aforementioned studies focusing only on b2c car sharing with reductions ranging from 11% to 51%. This suggests that the reductions from c2c sharing are less than from b2c sharing. One reason can be that a c2c rental car is much cheaper than renting a b2c car, which means that the mobility inducement effect of c2c sharing is higher than that of b2c sharing. Another reason may be that b2c cars are usually new, small and fuel-efficient, while cars shared through a c2c platform can be of any type and will, on average, be more polluting than b2c cars.

One of the general questions regarding the environmental impacts of the sharing economy concerns the extent of overall rebound effects [4]. As many goods now have become cheaper to rent than before (or even free), the real income of consumers increases. This additional income will, at least partially, be spent on other consumer goods. In particular, in home sharing and car sharing practices, users can realize substantial savings compared to the traditional alternatives (hotel, car rental). Another open question is whether the environmental benefits of sharing smaller items like clothes and toys outweigh the additional environmental cost of transporting and cleaning such goods [26].

## 5. Political economies and environmental futures

On the basis of the scant evidence of the environmental impact of the sharing economy, one can—tentatively—conclude that the environmental impacts of sharing are likely to be positive, but possibly much smaller than some claim and hope for. There is a clear need for a better understanding of the environmental impact of the sharing economy, its rebound effects, and how its impacts compare to the impacts of related business models (second-hand, product-service, and on-demand economy). Having said this, the environmental benefits of sharing are by no means a technological given and, as such, cannot be predicted accurately in advance. Impacts will crucially depend on the evolving business models and user practices, as well as the design of complementary institutions which are still under construction [1,3]. On top of this, industry analysts expect that the Internet-of-Things (IOT) will lead to yet another wave of new business models and service innovations with important consequences for the sharing economy [27]. Once many consumer goods have a permanent Internet connection, sensors and smart locks, sharing such goods through platforms becomes easier, cheaper and safer.

Given the uncertainty regarding institutional and technological changes to come, one can be sceptical about the validity of ‘point estimates’ about the future size and effects of the sharing economy. At this stage, claims regarding the future economic, social and environmental impacts remain highly speculative, for two reasons. First, platforms are reluctant to share usage data for scientific research or policy evaluation purposes. Hence, the little empirical data that is publicly available, is of poor quality. Platforms' reluctance to share data is understandable given their concerns about privacy and trade secrets. However, their stance is also strategic: by not granting researchers access to their data, the platforms can continue their claims about positive economic, social and environmental impacts of sharing while downplaying drawbacks, negative externalities and unintended effects [4]. Second, even if empirical data would become available for research purposes in the near future, any analysis of the future effects of sharing economy is necessarily based on past business models, user practices and institutional contexts. As a consequence, any predictions based on such an analysis will remain highly speculative.

To reflect on the future of the sharing economy nevertheless, I choose to articulate alternative governance regimes of the sharing economy, and the kinds of environmental and social effects that can be expected under each regime. I conceptualize three possible futures of the sharing economy based on how the political economy of sharing platforms will play out. As a first scenario, I foresee an extrapolation of the current neo-liberal development of sharing platforms. In this scenario, I assume that the existing platforms will integrate—technologically, organizationally and financially—into super-platforms. I call this scenario ‘platform capitalism’ [28], as it follows a market logic where commercial platforms continue to develop and integrate platform services in as many markets as possible to provide maximum convenience to mass of users. I call the second scenario ‘platform redistribution’, where governments tax property and redistribute rents from winners to losers. In this social-democratic future I also assume that governments regulate the sharing economy in a way that prosumers pursuing sharing activities are taxed in the same way as incumbent business to ensure fair competition. In a third scenario labelled ‘platform cooperativism’ [29], I foresee a more citizen-led wave of users who start their own platforms under democratic control.^5^

### (a) Platform capitalism

As a first scenario, one can envisage a further progression and integration of the current global platform companies. Currently, many platforms seem to follow this innovation strategy (especially, Amazon, Google, Uber and Airbnb). The possibility for service combinations are endless and facilitated by application programming interfaces that allow software components of different platforms to communicate effectively. One can think of a guest on a home sharing platform getting connected to a car, bike or boat sharing platform, or a host on a home sharing platform to a cleaning platform. What is more, sharing platforms would become ever more integrated in geographical databases, social media and payment systems. This development in itself does not imply a convergence of business models *per se*, but a first step towards integrated solutions in areas like mobility, lodging, food, care and education.

More advanced ways of integration happen when a single platform mixes multiple business models to provide a particular service. The scheme in figure 1 is again useful here. One example of integration that one can already witness is the rise of hybrid platforms where consumers can choose between c2c or b2c solutions, as with car sharing. Another example of convergence are the second-hand platforms who also started to offer the option for rent rather than the sole option to buy. Furthermore, b2c leasing services common in the product-service economy can be combined with sharing business models (allowing leasers to share the good with fellow consumers) and on-demand business models (allowing leasers to use the good to deliver a service). Such integration efforts provide consumers with maximum flexibility and variety of options while the platform acts as a ‘one-stop-shop’.

A second development underlying the capitalist scenario lies in the increased impersonality of c2c interaction via online platforms. Some have called this development in the sharing economy the trend from c2c to c2b2c [31]. In the latter type of platform, the platform mediates much more actively between two sharing consumers. Where platforms initially focused on matching supply and demand using an algorithm and payment system, platforms started to provide a host of other services including insurance, logistics, tax payment, cleaning, etc. This trend fits in the scenario of emerging super-platforms with many options and functionalities as to provide maximum conveniences for seamless consumption.

A third development concerns the advent of the IoT, which most analysts expect to materialize in new business models in the next decade [27]. Though, as for sharing economy, there is not a single definition of IoT, the key to this technological development is that most objects will have microchips, sensors and IP addresses so as to communicate object and environmental information to the Internet. One likely effect of IoT is that c2c sharing becomes easier as access to goods can be organized in a fully automated way. More specifically, once smart locks become ubiquitous, renters can be granted access to goods without the necessity of meeting the owner. The acclaimed benefits of sharing for social cohesion in neighbourhoods (as more strangers meet with the advent of sharing) would disappear again.

Another likely consequence of IoT is that shared goods can be monitored continuously during use using sensors, cameras and other devices. In this way user behaviour can be recorded and used for further service innovations and customization. At the same time, monitoring also allows parties to assess ‘proper use’ and, in case of accidents or theft, to provide digital evidence of improper behaviour. Not only will consumers who rent out their goods be interested in having the opportunity to monitor the use of their own goods, also insurance companies will experiment with new business models to tailor insurance based on object and user data, and to customize insurance price accordingly.

The convergence of business models, the trend towards c2b2c and the advent of IoT would all further professionalize the sharing economy and, as a consequence, also enforce the data position of platforms. These developments taken together will mean that from the receiving consumer side, services will become ever more one-stop-shop, seamless and just-in-time. Most likely, these innovations will be driven by global companies with existing ICT, big data and retail competencies, large installed user bases and supportive e-commerce legislation at national and transnational^6^ levels. If these companies were to grow and expand towards ever more markets, their sheer size and global nature would further reinforce the political power they already assume and display. This may imply that government policies regarding taxes and regulations will become ever more adapted to the needs and wishes of super-platforms. It is also likely that the current privacy laws that forbid platforms to hand over data to governments but allow them to sell data to other commercial parties will be continued. Finally, as for-profit platforms become ever more present and efficient in organizing peer-to-peer exchange, non-monetized sharing and offline sharing may well be substituted by monetized and online sharing platforms on a large scale. Only if for-profit platforms succeed in developing commercial business models based on peer-to-peer non-monetized transactions, which have been scarce so far, will super-platforms contribute to the further ‘economization’ of social life [1].

Although the sharing economy and related collaborative-consumption businesses are likely to grow rapidly in this scenario, the environmental benefits of a platform-capitalistic future remain highly uncertain. On the one hand, globally operating platforms will not be pressured neither by national governments nor by users to re-design their business models in an environmentally friendly manner. Indeed, platforms are profit-driven and shareholders will judge management accordingly. Furthermore, with the restrictive data access policies that platforms follow, it will be hard for scientists and politicians to build an evidence-base about environmental effects in the first place [4]. Such evidence is especially relevant to assess the aforementioned concerns about rebound effects.

On the other hand, the power of commercial platforms lies *inter alia* in their technological, managerial and marketing abilities to scale up business models by convincing mainstream consumers to join such platforms. With the further merger of the business models in super-platforms, supported by tailored insurance policies, tracking systems, and logistic services, it is likely that collaborative consumption quickly becomes a mass phenomenon. Hence, in so far as global for-profit platforms will generate positive environmental benefits, such benefits are likely be realized in the short term and at a mass scale.

### (b) Platform redistribution

In a second scenario, governments take up their traditional role of regulating innovation from a public interest and social justice perspective. Sharing platforms generate large welfare surplus as evidenced by the large volume of transactions. However, as most turnover is realized on home sharing platforms, the largest sums of income flow to home owners in popular (tourist) cities. Home owners in such cities are generally well off as they can afford to own a house in such cities in the first place. With consumers turning their consumer goods into capital assets on which serious returns are being made, capital owners profit most and inequality is expected to rise [34]. One can call this the ‘Piketty-effect’ of the sharing economy where returns on capital are much higher than returns on labour [4,35]. Note that this equally applies to owners of parking spaces, cars, campers, boats, aeroplanes, etc. What is more, inequality may be further spurred as rents for a regular apartment may go up with the value of houses, which increases in neighbourhoods where home sharing is common. As a consequence, the sharing economy does not only increase consumer welfare in absolute terms, but it also leads to a more unequal distribution of wealth among individuals.^7^

A classic governmental response to these developments is to increase the tax on capital held by consumers accordingly. This would not only entail higher tax on real estate property, but also on cars and parking space.^8^ Such tax proposals have historically been hard to realize for any political party, as home ownership and car ownership have become dominant middle-class values. Nevertheless, a political window is likely to emerge, in particular, in large cities where space and resources are scarce and cities look for new sustainability policies [3]. And car and home ownership may well go down as a larger part of the urban population can no longer afford it. This would mean that fewer voters may be inclined to vote against higher taxes on ownership. Sharing platforms also provide a good alternative for ownership. Hence, politicians proposing higher taxes on ownership would not exclude their (low-income) voters from the use of these goods, as they would have done so in the past.

Apart from taxes on property, governments can also tax the revenues that users make by sharing, especially from valuable property like houses and parking spaces. To the extent that such taxes are substantial, users may turn to alternative platforms using virtual currencies. Hence, the viability of shifting taxes also depends on the ability of governments to tax exchanges made in virtual currencies as well.

A new taxation system can make effective use of platforms to tax activities that previously were hard to monitor. Though at present most platforms seem reluctant to work together with governments, some platforms, such as Airbnb, already start collecting taxes for governments. The underlying tension that is to be resolved concerns the global interests and need for standardization of platforms operating from a particular territory (mostly, from the USA) and the national interests of governments outside this territory. The further development and integration of digital identity systems into platforms provides governments with a technological opportunity to track transactions of individual users and tax them accordingly. Such data would also enable local governments to defend other public interests, such as malpractices on second-hand platforms and nuisance caused by home sharing. It would further help to enforce regulatory caps, for example, on the number of days a resident is allowed to rent out a house, boat, or parking space [3].

This still leaves open the question if the same governments will also be able to claim tax on the profits that platforms make as a function of the volume of transactions in their respective territories. This question is especially pertinent in cases where platforms achieve near-monopoly positions, and, as a result, realize super-normal profit margins on their services. Redistribution of the gains from the sharing economy, thus, would include both the revenues of users and the revenues of platform shareholders.

In a scenario of redistribution by shifting taxation, the tax logic for on-demand platforms can also be adapted. In particular, freelancers doing gigs in sectors as taxi, cleaning, home delivery and cooking are currently treated as independent contractors by the platforms. In many cases, gig workers do not declare their earnings to the tax office, while the platforms do not grant the tax office access to their records so as to protect the privacy of their workers. Even if for many gig workers these earnings do not constitute their primary income—and earn little per hour anyway—the current situation is one of unfair competition between tax payers and tax avoiders. Three roads in the redistribution scenario can then be envisaged. First, platforms can charge taxes on their own and transfer these to the government in question as well as social insurances that better protect freelancers against sickness or work fluctuations. Such initiatives require freelancers to get organized, either in new types of unions or supported by unions who seek to enlarge their membership base from workers to freelancers. The latter development is nascent in Sweden where a white-collar union called Unionen negotiates with platforms for freelancers' rights and benefits [37]. A second solution would be to consider platform workers as platform employees given their strong dependence on the platform. This solution is in line with existing labour laws in many countries and currently underlies multiple law suits, especially against Uber. Thus income tax and social insurance have to be paid following standing collective labour agreements. A third, more radical, solution is to abolish tax for freelance services altogether by shifting the tax base entirely from service labour^9^ to consumer goods, housing, and earnings from capital investments. This will be a stimulus for reducing persistent unemployment under the less educated workforce. At the same time, higher taxes on material goods create more demand for repair and maintenance services, further contributing to sustainable development. Sweden's recent decision to lower tax on repair services is an early example [38].

Shifting taxes can achieve two goals in one: tax revenues can be used to redistribute returns from winners to losers, while sustainable consumption through sharing is stimulated if ownership gets more expensive compared to usage. Furthermore, an indirect effect may be that goods and houses will be redesigned to make them more suitable for sharing and longevity. At the same time, as governments get involved in taxing sharing and on-demand platform activities, they will also be able to construct their own databases and perform their own research on the impacts of sharing (and on-demand) platforms. On the basis of such analyses, government policies and tax rates can be adjusted and fine-tuned periodically.

### (c) Platform cooperativism

Though many expect commercial platforms to continue to dominate sharing economy at least in the short run, there is a recent movement that experiments with alternative forms of platform ownership and governance. These forms vary from crowdfunding and equity shares for users, to platforms erected by municipalities, consumer organizations or voluntary organizations, to cooperatives, and even blockchain-based platforms without ownership. Among these, the cooperative movement seems most active^10^ where ‘platformcoops’ are owned by users and some governance form is chosen to grant users a say into the governance and evolution of the platform [29,39].

A third scenario, then, can be conceptualized as driven by a cooperative movement that will be able to scale up or replicate their platforms supported by ideological motives and ICTs. Historically, cooperatives have proven to be very successful and also scalable in particular sectors in the economy, especially in insurance and agriculture. Hence, there is both a legal basis and organizational affinity with the cooperative form [1]. Furthermore, it should be remembered that many carsharing organizations are already run as cooperatives, especially in Germany [40].^11^ This would mean that such initiatives do not have to start from scratch, but may be able to leverage the knowledge and legitimacy of the cooperative form as it has emerged historically over centuries.

A key advantage of cooperatives is that returns for users can be much higher compared to for-profit platforms, as profits made by cooperatives are divided again among users [29]. In particular, users on platform cooperatives save on the fees charged by commercial platforms (generally 15–20%). Furthermore, users on platformcoops may also be able to circumvent government taxes using alternative currencies, or using no currency at all. In a platform cooperative setting, the monetization of sharing on platform is contingent: both monetized and non-monetized sharing models can in principle be supported by platform cooperatives. A final advantage for users is that they can retain ownership over the data that are generated by their actions. Privacy vis-à-vis both corporations and governments can be ensured, if so decided by users themselves.

In the current age of platforms, the cooperative form is mostly seen as a solution to worker exploitation in the on-demand economy, where platforms exercise control over workers as platforms can decide on participation based on reviews, with some like Uber also setting prices [29]. By having the workers owning and controlling the platform instead, exploitative practices can be avoided by agreeing on minimum wage, work times and insurances. Cooperatives have also been conceived as a model for sharing economy platforms. Here, two versions can be distinguished. The ‘old’ version where the cooperative owns the assets (as in most car sharing platformcoops), and a ‘new’ version where only the platform software and data are collectively owned and managed, while the goods being shared are individually owned and rented out.^12^ We also witness the cooperative model emerging in second-hand markets as an alternative to the for-profit business model of Ebay. In particular, the Germany-based cooperative Fairmondo provides a second-hand marketplace and has become active in other countries as well.

Some, though, have argued that running a platformcoop is a challenging venture [41--43]. It remains to be seen if cooperatives can effectively make use of and contribute to the ongoing technological developments that are foreseen. They have a limited ability to raise venture capital and to do R&D on their own, which severely limits their capacity to engage in innovation and software development. Furthermore, as users may have divergent interests depending on their involvement (part-time versus full-time) and views on remuneration for their efforts (voluntary or paid), the management of a fast-growing platform tends to be accompanied by personal conflicts and ideological oppositions. Such tensions are not easily resolved, in particular, if one views the platform not only as a form collective ownership, but also as a way to exercise democratic control. In the German car sharing sector, for example, most cooperatives have remained small while for-profit platforms have successfully scaled up [40]. And those cooperatives that scale up tend to change their organizational structure along the way [44].

While for-profit platforms currently exploit ICTs very well to their advantage, the cooperative model can also make use of ICTs to scale up its model. The collective governance of large platformcoops can be supported by new online discussion tools, management systems and payment systems. It should further be remembered that transactions over sharing platforms (as well as second-hand, product-service and on-demand platforms) are predominantly local, home sharing being the exception. Hence, scaling up a cooperative platform is not a necessity for its viability as long as it satisfies a local user base. Instead, the cooperative model can diffuse by replication instead of by scaling. Particularly, with the use of open source software, local initiatives can benefit from platform architectures tested elsewhere.^13^

Though cooperatives would enhance the social sustainability of sharing economy platforms, the environmental effects would not necessarily be fundamentally different from commercial platforms. Cheaper access would induce more consumption and indirect rebound effects can still be large. Yet, analogous to farmer cooperatives, consumer cooperatives may also be used for joint purchase of consumer goods. The latter may stimulate environmental consumption substantially, because individual purchase decisions by a large number of individuals can then be coordinated. This does not only mean that users can negotiate lower prices through their cooperative platform, but they can also kick-start the adoption of new and sustainable technologies (e.g. electric vehicles or recyclable designs). More generally, cooperatives can avoid the classic lock-in problem that individual consumers face when confronted with a sustainable technology. Cooperatives provides an organizational form to overcome this coordination problem.

### (d) Scenarios compared

The three futures just articulated are quite distinct in their dominant institutional logic, enabling technology and spatial scale. As a summary, table 1 lists these aspects for each scenario. Nascent examples of different countries are also highlighted. A differentiated pattern may already be visible when comparing the USA as a typical Anglo-Saxon free-market economy, Sweden as a highly unionized country, and Germany with its cooperative tradition.

**Table 1.** The three scenarios compared.

platform scenario	institutional logic	enabling technology	typical scale	nascent examples
capitalism	market	Internet-of-Things	global	United States
redistribution	state	digital identity systems	national	Sweden
cooperativism	community	open source software	local	Germany

However, even within each country, different scenarios may coexist. Today, with car sharing business models varying with urban densities, we witness for-profit car manufacturers operating in large metropolitan areas and cooperatives providing their services in smaller towns [40]. Furthermore, in the future, we may see a sectoral differentiation with some sectors fully organized by global super-platforms (e.g. driverless carsharing), other sectors becoming heavily taxed and regulated by government (e.g. home sharing for tourists), and yet other sectors becoming dominated by cooperatives (e.g. on-demand labour sectors).

Finally, the dominant spatial scale at which the political economy of platforms will play out ranges from the global in the capitalist scenario, to the national in the redistribution scenario, and to the local in the cooperativist scenario. The question which scenario will emerge is to an important extent a question about subsidiarity [33, p. 94]. Indeed, some may argue for local regulations given that platforms organize local marketplaces, others for national regulations given that platforms often touch nationally regulated professions and sectors, or yet others for transnational (e.g. European) regulations given the existing policies regarding digital markets, consumer protection, and technological standardization. While current platforms plea for transnational harmonization and market integration given the inherent efficiency gains, the societal costs and benefits will be experienced mostly at the local level**.**

## 6. Conclusion

The key takeaway from this essay holds that the environmental and social effects of the sharing economy will depend most importantly on institutional changes still to come. Institutions do not only regulate activities on sharing platforms, but also shape the future development of the socio-technical infrastructure that emerges as the sharing economy scales up. I have presented three future political economies: a market-led future cumulating in super-platforms, a government-led future that shifts taxation from labour to capital, and a citizen-led future based on cooperatively owned platforms under democratic control. The three futures will not just differ in the distribution of economic gains and political power in an economy dominated by online platforms for sharing goods and on-demand labour, but also in the size and nature of the environmental impacts that can be expected to occur.

## Footnotes

1 Note that the definition refers to physical assets, and thus includes information goods on a physical carrier like books and DVDs, but excludes digital files which can be copied just by posting them online.

2 Note that this reasoning is consonant with Polanyi's [8] substantive view on the economy.

3 In economic theory, the rapid growth of sharing can be explained by the reduction of ‘transaction costs’ by the advent of online platforms. Sharing among strangers used to involve such high costs associated with searching and contracting that most people did not engage in stranger sharing at all. With the advent of Internet, transaction costs have gone down substantially and, as a consequence, many online marketplaces for sharing have emerged.

4 The distinction here between ridesharing and ride-hailing follows the Associated Press Stylebook: ‘Ride-hailing services such as Uber and Lyft let people use smartphone apps to book and pay for a private car service or in some cases, a taxi. They may also be called ride-booking services. Do not use ride-sharing’ [7]. See also Meelen and Frenken [17].

5 A fourth scenario that I will not comment on, is the possibility that the sharing economy will remain small as it may become discredited due to moral or regulatory resistance or may simply fail to continue to attract investment and revenue (see also [30]).

6 See the vision on the sharing economy outlined by the European Commission [32] as well as a further discussion by Hatzopoulos & Roma [33].

7 For a different view, see Sundararajan [27] who views the sharing economy as contributing to ‘inclusive growth’ by providing low-income groups with new opportunities.

8 Concerning parking, there seems to be room for tax increase as current prices of parking for residents tends to be far below market prices [36].

9 This would include services needed to repair, redesign or recycle as further stimulus for sustainable consumption.

10 See http://platformcoop.net/ and http://wiki.p2pfoundation.net/Platform_Cooperativism.

11 For example, Münzel *et al*. [40] found that in Germany more than half of the car sharing organizations are cooperatives.

12 As, for example, the Amsterdam-based initiative called FairBnB which develops an alternative to home sharing platform Airbnb.

13 In the future, replication can also entail the design of shared material goods with the use of open source hardware and 3D printing [45].
